# 消融术和亚肺叶切除术治疗Ia期非小细胞肺癌患者疗效的对比

**DOI:** 10.3779/j.issn.1009-3419.2021.104.09

**Published:** 2021-09-20

**Authors:** 恒 赵, 坤 范, 泓懿 王, 博豪 刘, 益行 李, 润仪 陶, 芝馀 王, 佳 张, 军科 付, 广健 张

**Affiliations:** 710061 西安，西安交通大学第一附属医院 The First AffilIated Hospital of Xi'an Jiaotong University, Xi'an 710061, China

**Keywords:** 消融术, 亚肺叶切除术, 肺肿瘤, 总生存期, Ablation, Sub-lobotomy, Lung neoplasms, Overall survival

## Abstract

**背景与目的:**

肺癌是我国死亡率最高的肿瘤，不同治疗方式对于患者预后的影响具有重要的意义。通过比较消融术和亚肺叶切除术治疗Ia期非小细胞肺癌（non-small cell lung cancer, NSCLC）患者的生存期，分析该两种治疗方案对早期肺癌患者预后生存的不同影响。

**方法:**

运用美国国立癌症研究所监测、流行病学和结果数据库（The Surveillance, Epidemiology, and End Results, SEER），我们从2004年1月-2015年12月之间筛选了符合条件的Ia期NSCLC患者进行研究。通过倾向性评分匹配，筛选出228例采用消融术治疗和228例采用亚肺叶切除术治疗的Ia期NSCLC的患者，并进行*Kaplan-Meier*生存曲线分析。比较Ia期NSCLC患者在经过匹配和调整后接受不同治疗方式的总生存率情况。

**结果:**

*Kaplan-Meier*生存曲线分析消融组和亚肺叶切除术组之间的生存曲线存在明显差异（*P* < 0.05）。单因素分析中，与消融组相比，亚肺叶切除术的风险比（hazard ratio, HR）为0.571（95%CI: 0.455-0.717），即采用亚肺叶切除术治疗的患者产生的不良结局风险是消融术的0.571倍；在多因素分析中，亚肺叶切除术的HR为0.605（95%CI: 0.477-0.766），即采用亚肺叶切除术治疗的患者产生的不良结局风险是消融术的0.605倍。以上结果均提示亚肺叶切除术治疗Ia期NSCLC患者的总生存率高于消融术。

**结论:**

使用消融术和亚肺叶切除术治疗的Ia期NSCLC患者的总生存率之间存在明显差异。采用亚肺叶切除术治疗Ia期NSCLC的患者总生存率明显高于消融术组。

## Abstract (en)

**Background and objective:**

Lung cancer has the highest mortality in China. Different treatments are of great significance to the prognosis of patients. By comparing stage Ia non-small cell lung cancer (NSCLC) patients' survival rates for ablation and for sub-lobectomy, we studied the difference in the effects of the two treatments on patient prognosis.

**Methods:**

Using the Surveillance, Epidemiology, and End Results (SEER) database, we screened eligible patients with stage Ia NSCLC from January 2004 to December 2015. Then, 228 patients treated with ablation and 228 patients treated with sub-lobotomy were then selected based on propensity score matching. After stratification, matching, and adjustment the *Kaplan-Meier* analysis was performed to compare the overall survival rates of patients treated with the two procedures.

**Results:**

The *Kaplan-Meier* survival analysis showed that there is a significant difference between the ablation group and the sub-lobectomy group (*P* < 0.05). In the univarlable analysis, the hazard ratio (HR) of sub-lobotomy group was 0.571 (95%CI: 0.455-0.717) compared with the ablation group. Patients treated with sub-lobectomy had a 0.571 times greater risk of adverse outcomes than those treated with ablation. In the multivariable analysis, the HR for sub-lobectomy group was 0.605 (95%CI: 0.477-0.766) compared with the ablation group. Patients treated with sub-lobectomy had a 0.605 time greater risk of adverse outcomes than those treated with ablation. The results suggested that the overall survival rate of patients with stage Ia NSCLC treated with sub-lobotomy was higher than that of patients treated with ablation.

**Conclusion:**

This study suggests that there is a significant difference in overall survival of stage Ia NSCLC patients treated with ablation and with sub-lobotomy. Patients treated with sub-lobotomy for stage Ia NSCLC had higher overall survival than those treated with ablation.

目前，肺癌仍是全球发病率最高的肿瘤之一。根据美国癌症中心的数据，2019年约有228, 150例新诊断的肺癌患者，其中80%-85%以上患有非小细胞肺癌（non-small cell lung cancer, NSCLC），有142, 670例患者死于肺癌^[1-3]^。通过美国癌症联合委员会I期或II期的疾病诊断标准，早期肺癌患者占新诊断病例的16%^[4]^。目前，治疗Ia期NSCLC的金标准是手术治疗加淋巴结清扫^[5]^。其中，亚肺叶切除术是常用的手术方式，主要包括肺楔形切除和肺段切除术。然而在实际情况中，超过20%的早期NSCLC患者因为年龄、肺功能严重受损或其他合并症而不能进行手术^[6]^。因此，非手术治疗作为一种新兴的治疗手段，对于不能耐受手术的NSCLC患者提供了有效的治疗方案。消融术是在影像引导下，通过激光、冷冻或电灼术等方法，对局部肿瘤进行摧毁的非手术治疗。该种方法对不能耐受手术的NSCLC患者取得了良好的局部控制效果^[7, 8]^。并且，消融术联合化疗等治疗方法，相对于单一化疗而言，疗效确切，有效率高，生存时间久，为NSCLC患者提供更多治疗方案^[9]^。到目前为止，还没有关于运用大规模癌症数据库对消融术和亚肺叶切除术治疗早期NSCLC患者生存期的影响的研究。此外，目前很少有研究来评估这两种治疗方法的有效性。本研究是运用美国国立癌症研究所（National Cancer Institute, NCI）监测、流行病学和结果数据库（The Surveillance, Epidemiology, and End Results, SEER）中的数据，比较接受消融术和亚肺叶切除术的Ia期NSCLC患者的总生存率情况，以评价两种治疗方法的效果差异。

## 1 方法

### 1.1 数据来源

本研究的数据是从SEER数据库中提取的，SEER由NCI于1973年建立，是北美最具代表性的大型肿瘤登记注册数据库之一。其收集了大量循证医学的相关数据，为临床医师的循证实践及临床医学研究提供了系统的证据支持和宝贵的第一手资料^[10]^。SEER数据库包含患者的基本信息、发病部位、肿瘤大小、病理类型、生存状态、存活时间和治疗手段等内容，包含大约10%的美国人口的数据。

### 1.2 研究人群

在本项研究中，我们筛选出了从2004年1月-2015年12月Ia期NSCLC患者，即肿瘤直径≤3 cm、不伴有区域淋巴结转移和远处转移的患者。所有患者均只接受过消融术治疗或亚肺叶切除术治疗，且患者术后病理均证实为NSCLC。其中消融术包括射频消融、微波消融、冷冻消融及激光消融等疗法，亚肺叶切除术包括肺楔形切和肺段切除。SEER数据库中记录有患者的具体情况以及治疗方式等信息。

### 1.3 基本信息

研究病例的基线包括以下9种：年龄段、性别、人种、肿瘤大小、肿瘤位置、肿瘤分级、组织学类型、肿瘤偏侧性和诊断年份。

### 1.4 病理资料

SEER数据库中包含了所有筛选患者的病理类型，均以ICD-O-3形态学编码的形式记录。根据组织病理类型，所有NSCLC（8046/3）包括：①鳞状细胞癌（ICD-O-3形态学编码：8052/3、8070/3、8071/3、8072/3、8073/3、8074/3、8076/3、8083/3、8084/3、8123/3）；②肺腺癌（ICD-O-3形态学编码：8140/3、8141/3、8200/3、8244/3、8250/3、8251/3、8252/3、8253/3、8254/3、8255/3、8260/3、8263/3、8290/3、8310/3、8323/3, 、8333/3、8470/3、8471/3、8480/3、8481/3、8490/3、8550/3、8551/3、8574/3）；③大细胞肺癌（ICD-O-3形态学编码：8012/3、8013/3、8014/3）；④其他（ICD-O-3形态学编码：8022/3、8030/3、8031/3、8032/3、8033/3、8050/3、8082/3、8201/3、8230/3、8246/3、8249/3、8430/3、8507/3、8560/3、8720/3）。根据我们筛选的标准，入选的所有Ia期NSCLC的患者全部接受消融治疗或亚肺叶切除，手术方式也均以代码的形式记录。其中消融术包括：激光消融或冷冻消融（SSER手术代码12）、电灼消融（SEER手术代码13）；亚肺叶切除术包括：肺楔形切（SEER手术代码21）；肺段切除术（SEER手术代码22）。

### 1.5 统计分析

所有的数据均使用IBM SPSS 24.0版本进行分析。倾向评分匹配用于控制纳入病例的基线特征和潜在的差异；*Kaplan-Meier*分析用于比较消融组和亚肺叶切除组的生存曲线；*Cox*回归分析用于评估多因素对于Ia期NSCLC患者的总生存率的影响。

## 2 结果

### 2.1 研究队列的基线特征

我们通过倾向性评分匹配中的最邻近匹配法，设置卡钳值为0.03，从数据库中共筛选出了共456例在年龄、性别、人种、肿瘤大小、分级和组织学类型等方面情况相一致的患者，且经卡方检验，以上变量在消融组和亚肺叶切除组之间均无明显差异（*P*>0.05）。表 1显示了两组患者的基线特征。此外，为了验证我们所得结论的真实可靠性，我们还运用了最邻近匹配中的不同比例匹配、最优匹配等不同的倾向性评分匹配方法，对两组患者的生存结局进行分析，结果表明各种不同的匹配方法所得结论相一致（不同匹配方法见补充材料）。

**表 1:** 消融术和亚肺叶切除术的Ia期NSCLC患者的基线特征[*n*(%)] Baseline characteristics of patients with stage Ia NSCLC treated with ablation and sub-lobectomy [*n*(%)]

Characteristics	Total number	Ablation	Sub-lobectomy	*P*
Age (yr)				0.048
≥40, < 50	6 (1.32)	3 (1.32)	3 (1.32)	
≥50, < 60	23 (5.04)	10 (4.39)	13 (5.70)	
≥60, < 70	118 (25.88)	63 (27.63)	55 (24.12)	
≥70, < 80	181 (39.69)	83 (36.40)	98 (42.98)	
≥80	128 (28.07)	69 (30.26)	59 (25.88)	
Gender				< 0.001
Male	191 (41.89)	107 (46.93)	84 (36.84)	
Female	265 (58.11)	121 (53.07)	144 (63.16)	
Race				0.169
White	391 (85.75)	198 (86.84)	193 (84.65)	
Black	33 (7.24)	19 (8.33)	14 (6.14)	
Asian	31 (6.80)	10 (4.39)	21 (9.21)	
Others	1 (0.22)	1 (0.44)	0 (0.00)	
Tumor size (cm)				0.264
≤1	61 (13.38)	26 (11.40)	35 (15.35)	
>1, ≤2	257 (56.36)	126 (55.26)	131 (57.46)	
>2, ≤3	138 (30.26)	76 (33.33)	62 (27.19)	
Tumor location				< 0.001
Main bronchus	1 (0.22)	1 (0.44)	0 (0.00)	
Upper lobe	277 (60.75)	140 (61.40)	137 (60.09)	
Middle lobe	22 (4.82)	14 (6.14)	8 (3.51)	
Lower lobe	150 (32.89)	69 (30.26)	81 (35.53)	
Others	6 (1.32)	4 (1.75)	2 (0.88)	
Grade				< 0.001
Ⅰ	64 (14.04)	28 (12.28)	36 (15.79)	
Ⅱ	89 (19.52)	40 (17.54)	49 (21.49)	
Ⅲ	70 (15.35)	36 (15.79)	34 (14.91)	
Ⅳ	3 (0.66)	0 (0.00)	3 (1.32)	
Unknown	230 (50.44)	124 (54.39)	106 (46.49)	
Histologic type				0.004
Squamous cell carcinoma	98 (21.49)	65 (28.51)	33 (14.47）	
Adenocarcinoma	284 (62.28)	123 (53.95)	161 (70.61)	
Large cell carcinoma	5 (1.10)	0 (0.00)	5 (2.19)	
Others	69 (15.13)	40 (17.54)	29 (12.72)	
Laterality				0.370
Left	215 (47.15)	102 (44.74)	113 (49.56)	
Right	241 (52.85)	126 (55.26)	115 (50.44)	
Year of diagnosis				0.052
2004-2006	106 (23.25)	46 (20.18)	60 (26.32)	
2007-2009	144 (31.58)	76 (33.33)	68 (29.83)	
2010-2012	105 (23.03)	60 (26.32)	45 (19.74)	
2013-2015	101 (22.15)	46 (20.18)	55 (24.12)	
Total	456	228	228	
NSCLC: non-small cell lung cancer				

经过*Kaplan-Meier*分析显示，在年龄（*P*=0.048）、性别（*P* < 0.001）、肿瘤位置（*P* < 0.001）、肿瘤分级（*P* < 0.001）和组织学类型（*P*=0.004），两组之间的总生存率（overall survival, OS）之间存在显著性差异。而在人种（*P*=0.169）、肿瘤大小（*P*=0.264）、偏侧性（*P*=0.370）和诊断年份（*P*=0.052），两组之间没有观察到显著性差异（表 1）。

### 2.2 比较消融组和亚肺叶切除术组的疾病死亡率和中位生存期

Ia期NSCLC患者的总体特异性死亡率为68.20%（311/456），消融组和亚肺叶切除组的死亡率分别为76.32%（174/228）和60.09%（137/228）。Ia期NSCLC患者的平均生存时间为64.68个月，消融组和亚肺叶切除组的平均生存时间分别为51.92个月和76.72个月。Ia期NSCLC患者的总中位生存期为50个月，消融组和亚肺叶切除组的中位生存期分别为37个月和69个月（表 2）。与消融组相比，Ia期NSCLC患者亚肺叶切除组的风险比（hazard ratio, HR）为0.571（95%CI: 0.455-0.717）（表 3）。

**表 2:** 消融组和亚肺叶切除组的死亡率、中位生存期和平均生存时间 Association with cancer-specific mortality, median survival time and mean survival time among patient groups

Groups	Mortality (%)	Median survival time (mon)	Mean survival time (mon)
Overall	68.20	50	64.68
Ablation	76.32	37	51.92
Sub-lobectomy	60.09	69	76.72

**表 3:** 单因素分析消融组和亚肺叶切除组患者的存活率 Univariate analysis for survival in ablation and sub-lobectomy groups

	*n*	Hazard ratio	95%CI	*P*
Ia NSCLC	456	0.571	0.455-0.717	< 0.001
Squamous cell carcinoma	98	0.818	0.510-1.312	0.405
Adenocarcinoma	284	0.584	0.432-0.789	< 0.001

### 2.3 *Kaplan-Meier*分析比较两组生存曲线

在*Kaplan-Meier*分析中，我们观察到运用消融术和亚肺叶切除术治疗Ia期NSCLC患者的生存曲线之间存在明显差异，见图 1。在Ia期NSCLC患者中，消融组和亚肺叶切除组之间的生存率存在明显差异（*P* < 0.001）。采用亚肺叶切除术的Ia期NSCLC患者的生存率高于消融组。另外，我们发现在Ia期鳞癌的患者中，消融组和亚肺叶切除组之间的生存曲线没有显著性差异（*P*=0.397），见图 2；而在Ia期腺癌的患者中，消融组和亚肺叶切除组之间的生存曲线存在明显差异（*P* < 0.001），见图 3。提示在患有腺癌的患者当中，采用亚肺叶切除术的Ia期NSCLC患者的生存率高于消融组。

**图 1:**
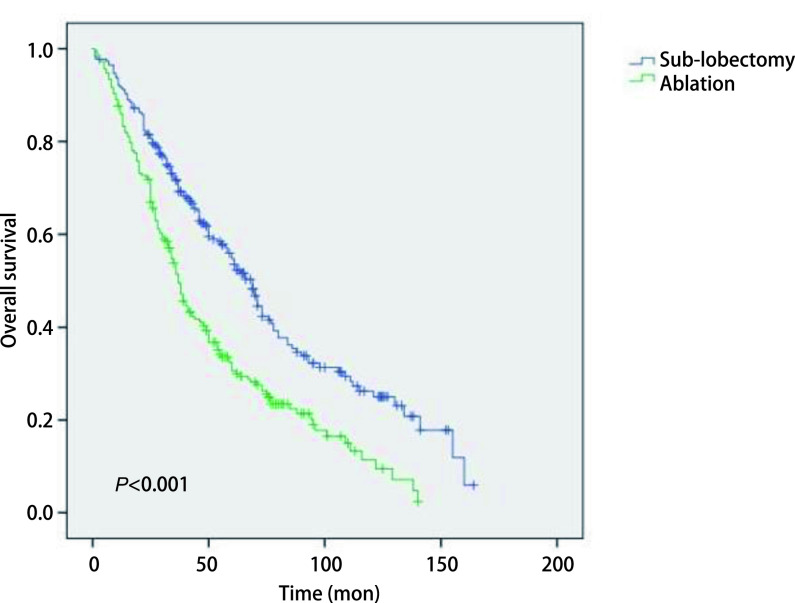
Ia期NSCLC患者接受消融治疗和亚肺叶切除治疗后*Kaplan-Meier*生存曲线比较 Comparison of *Kaplan-Meier* survival survival curve of stage Ia NSCLC parients after sub-lobectomy and ablation treatment

**图 2:**
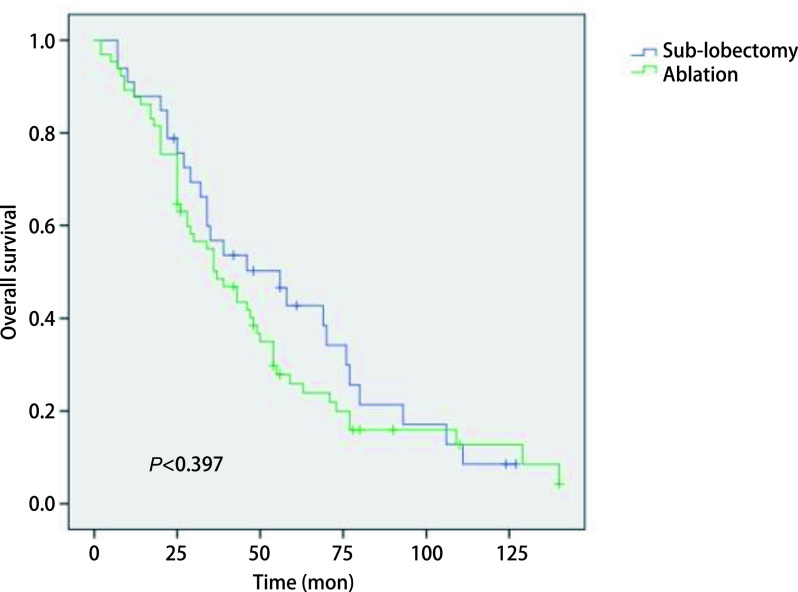
鳞癌患者接受消融治疗和亚肺叶切除治疗后*Kaplan-Meier*生存曲线比较 Comparison of *Kaplan-Meier* survival survival curve of squamous cell carcinoma patients after sub-lobectomy and ablation treatment

**图 3:**
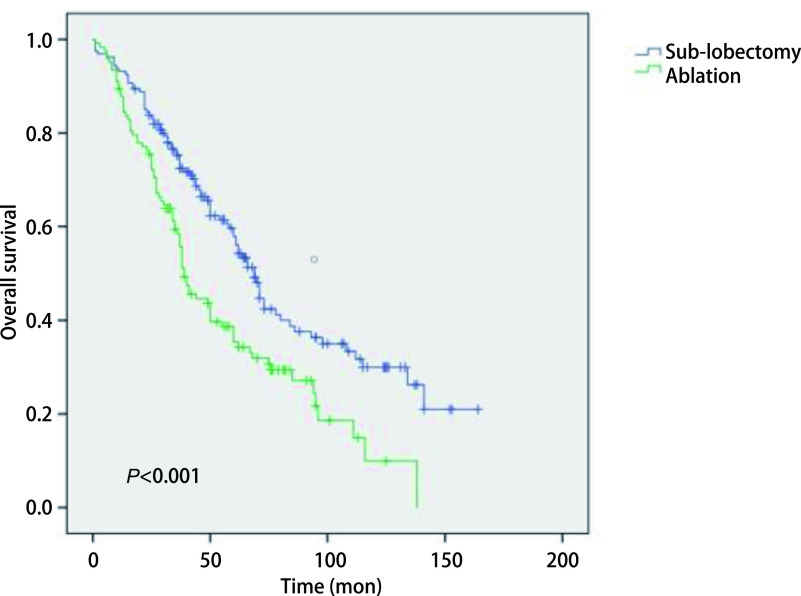
腺癌患者接受消融治疗和亚肺叶切除治疗后*Kaplan-Meier*生存曲线比较 Comparison of *Kaplan-Meier* survival curve of adenocarcinoma patients after sub-lobectomy and ablation treatment

### 2.4 *Cox*比例风险回归分析消融组和亚肺叶切除组患者的生存率影响

用单因素分析消融组和亚肺叶切除组患者的生存率，两组之间存在明显差异（*P* < 0.001）。与消融组相比，亚肺叶切除组治疗Ia期NSCLC患者的HR为0.571，即采用亚肺叶切除组治疗的风险是消融组的0.571倍。此外，在Ia期鳞癌的患者中，采用消融术和亚肺叶切除术对患者所面临的风险没有显著性差异，在腺癌的患者中，与消融组相比，亚肺叶切除组治疗的风险比为0.584。

在*Cox*风险比例模型中，我们首先分析了10个协变量：年龄、性别、人种、肿瘤大小、肿瘤位置、肿瘤分级、组织学类型、偏侧性、诊断年份和治疗方式。分析结果显示，与消融组相比，亚肺叶切除组的HR为0.573（95%CI: 0.448-0.733）（*P* < 0.001）（表 4），在*Cox*风险比例模型中，可以认为两种治疗方式对Ia期NSCLC患者的生存时间产生不同的影响，消融组和亚肺叶切除组之间存在显著性差异。

**表 4:** *Cox*风险比例模型 The *Cox* proportional hazards model

Covariate	HR	95%CI	*P*
Age (yr)			0.665
≥40, < 50	Reference		
≥50, < 60	0.620	0.164-2.343	0.481
≥60, < 70	0.821	0.246-2.744	0.748
≥70, < 80	0.820	0.245-2.738	0.747
≥80	0.947	0.280-3.202	0.930
Gender			0.002
Male	Reference		
Female	0.697	0.553-0.878	
Race			0.182
White	Reference		
Black	0.655	0.411-1.045	0.076
Asian	0.733	0.436-1.232	0.241
Others	1.953	0.262-14.574	0.514
Tumor size (cm)			0.974
≤1	Reference		
>1, ≤2	1.022	0.697 -1.500	0.911
>2, ≤3	1.045	0.694 -1.573	0.834
Tumor location			0.017
Main bronchus	Reference		
Upper lobe	0.037	0.004-0.307	0.002
Middle lobe	0.036	0.004-0.320	0.003
Lower lobe	0.045	0.005-0.376	0.004
Others	0.025	0.002-0.283	0.003
Grade			0.002
Ⅰ	Reference		
Ⅱ	1.571	0.975-2.533	0.064
Ⅲ	2.731	1.657-4.503	0.001
Ⅳ	1.737	0.403-7.492	0.459
Unknown	1.732	1.118-2.683	0.014
Histologic type			0.635
Squamous cell carcinoma	Reference		
Adenocarcinoma	0.912	0.686-1.212	0.524
Large cell carcinoma	1.774	0.579-5.441	0.316
Others	0.985	0.682-1.424	0.936
Laterality			0.330
Left	Reference		
Right	0.888	0.698-1.128	
Year of diagnosis			0.145
2004-2006	Reference		
2007-2009	0.907	0.678-1.212	0.509
2010-2012	0.756	0.539-1.061	0.106
2013-2015	0.646	0.422-0.989	0.044
Treatment			< 0.001
Ablation	Reference		
Sub-lobectomy	0.573	0.448-0.733	

之后，我们对上一个*Cox*风险比例模型进行了进一步的调整，排除了人种、肿瘤位置、偏侧性和诊断年份这些与临床关系不是非常密切的变量，并根据年龄、性别、肿瘤大小、分化程度、组织学类型和治疗方式重新调整*Cox*风险比例模型（表 5）。在该模型中，与消融组相比，亚肺叶切除组治疗Ia期NSCLC患者的HR为0.605（*P* < 0.001），即亚肺叶切除术组的风险是消融组的0.605倍（95%CI: 0.477-0.766），可以认为两种治疗方式对Ia期NSCLC患者的生存时间产生不同的影响，消融组和亚肺叶切除组之间存在显著性差异。可以认为相比于消融术，亚肺叶切除术有更好的生存预后。

**表 5:** 调整后的*Cox*风险比例模型 The adjusted *Cox* proportional hazards model

Covariate	HR	95%CI	*P*
Age (yr)			0.285
≥40, < 50	Reference		
≥50, < 60	0.510	0.139-1.877	0.311
≥60, < 70	0.738	0.227-2.400	0.613
≥70, < 80	0.777	0.239-2.526	0.674
≥80	0.922	0.282-3.022	0.894
Gender			0.007
Male	Reference		
Female	0.730	0.581-0.917	
Tumor size (cm)			0.923
≤1	Reference		
>1, ≤2	1.079	0.739-1.574	0.693
>2, ≤3	1.058	0.707-1.583	0.783
Grade			0.004
Ⅰ	Reference		
Ⅱ	1.559	0.975-2.492	0.063
Ⅲ	2.535	1.560-4.118	< 0.001
Ⅳ	1.828	0.423-7.910	0.419
Unknown	1.764	1.148-2.712	0.010
Histologic type			0.501
Squamous cell carcinoma	Reference		
Adenocarcinoma	0.890	0.672-1.178	0.415
Large cell carcinoma	1.910	0.629-5.804	0.254
Others	0.965	0.671-1.389	0.850
Treatment			< 0.001
Ablation	Reference		
Sub-lobectomy	0.605	0.477-0.766	

## 3 讨论

早期的NSCLC的标准治疗是手术治疗加淋巴结清扫^[11, 12]^，常规的手术治疗包括肺楔形切除术和肺段切除术。但是在实际情况中，相当多的患者因为年龄、肺功能不良以及心脏合并症等因素成为不可手术或高风险手术人群^[13, 14]^，该人群将面临术后重大风险。因此，近些年，例如消融术、立体定向放射治疗（stereotactic body radiation therapy, SBRT）^[15]^等非手术治疗的方法越来越多的应用于临床，为高危的不能进行手术的患者提供了可选择的治疗方案，对于部分患者，消融术甚至可以达到手术的效果。一项研究^[16]^比较了接受SBRT治疗（*n*=15）、手术治疗（*n*=10）、热消融治疗（*n*=6）或仅接受化疗（*n*=5）的患者在治疗后90 d内局部复发率和3级或3级以上不良事件的发生率，该研究报告SBRT治疗后的发生率为7%，手术治疗后为40%，热消融治疗后为0%，仅接受化疗后为40%。毕竟采用手术治疗的方式对于机体在一定程度上是一种创伤，相较于非手术治疗，它对患者的基础情况就提出了更高的要求，这也是部分高危患者采用非手术治疗的原因。实际上，消融术作为一种新兴的治疗手段，已经应用于临床治疗之中。相对于传统的标准疗法，射频消融术具有重要的优势^[17]^。在一项多中心实验中，早期肺癌经初次消融治疗之后，第12个月和第24个月的疗效分别为85.1%和77.2%，经过第二次治疗后，第12个月和第24个月的疗效增长至91.1%和84.4%^[18]^。射频消融术的一个主要优点是，它可以破坏肿瘤，而不会对周围的正常软组织造成重大损害^[19]^。一项研究^[20]^结果表明，射频消融术的安全性与经皮图像引导肺活检术相似。此外，有研究^[21]^证明了射频消融术是一种很有前途的肺部恶性肿瘤的治疗方法，具有令人满意的肿瘤破坏效果，尤其是直径≤3.0 cm的肿瘤。许多研究者^[22, 23]^认为肺射频消融术治疗不能切除的Ia期NSCLC是安全可行的。在单侧肺切除而对侧肺复发时，消融术已证明其对无手术死亡的单肺患者的安全性和有效性^[24, 25]^。综上所述，考虑到风险和益处，射频消融术作为一种微创策略，对于合并内科疾病的患者来说可能是一种非常有吸引力的治疗选择。两项证据等级为三级的前瞻性研究评估了消融术在Ia期NSCLC中的疗效。美国外科医师学会肿瘤学小组报告了消融治疗1年后的生存率高达91.67%^[26]^。在另一项研究^[27]^中，1年时局部控制率为84.4%（95%CI: 67.2-95.7），3年时为81.2%（95%CI: 54.3-95.9）。在射频消融术后常规放射治疗在不能手术的Ia期NSCLC患者中是可行的，并发症较少，无重大毒性^[28]^。然而在实际中，消融术治疗的患者也存在术后并发症，其中以自限性气胸最为常见，大部分患者均可以得到缓解，只有约10%-30%的患者需要进行胸腔闭式引流治疗^[29, 30]^，此外，其他罕见并发症也是存在的，例如肺出血^[31, 32]^、空气栓塞^[33, 34]^、胸腔积液^[35]^、支气管胸膜瘘^[36]^、支气管痉挛^[7]^，甚至死亡^[19]^等。尽管发生率较低，但是也为消融术治疗Ia期NSCLC患者的生存风险提供了可能。

根据我们的研究结果，采用消融术和亚肺叶切除术治疗均对Ia期NSCLC的患者的预后产生有利的影响。与消融术相比，采用亚肺叶切除术的患者的生存率更高，提示亚肺叶切除术优于消融术治疗。亚肺叶切除术在一定程度上也保留了肺功能，使患者的生存质量得到了提高。即只要患者条件允许，手术治疗是首选的，因为它具有更高的疾病控制率和生存率。对于那些不能耐受手术的Ia期NSCLC患者，消融术治疗也是有效的^[37]^，第八版NCCN指南也提出该项观点^[5]^。Kim等^[38]^曾根据年龄、性别、肿瘤淋巴结转移阶段和治疗时间，比较了射频消融术（*n*=8）与肺叶切除术和全肺切除术（*n*=14）治疗Ia期NSCLC患者。他们获得的5年生存率分别是：射频消融术25%和手术67%。此外，另一个关键点必须强调：淋巴结取样可以在手术干预时进行，从而可以识别未知的淋巴结发生转移情况和更精确的临床病理分期。尽管射频消融术的疾病复发率较高，但由于其低侵入性、可重复性、低并发症和短住院时间，应被视为无法手术患者的有效选择^[37]^。这对于Ia期NSCLC患者治疗方式的选择有一定的指导意义。

然而，本研究也存在着一些不足之处。虽然SEER数据库为我们提供了一个重要的大规模的数据收集平台，但是还是存在一定的局限性。首先，我们采用多种倾向性匹配法对队列进行了精确的匹配，尽可能的使两组之间的构成比上保持一致，但是本项研究实质上还是回顾性的研究，存在一定程度的不可避免的信息偏倚，使得数据的完整性并不完善。其次，SEER数据库仅提供了治疗的方式，而对于详细的治疗操作未进行记录，例如消融术是经皮或是经支气管镜等，这也是今后研究有待完善的领域。最后，我们还缺乏自己的原始研究数据，可以在掌握一定临床数据的基础上进行前瞻性的实验设计，相较于利用数据库进行回顾性研究，更有说服力。

通过我们对以上456例Ia期NSCLC患者的研究分析，结果表明：与消融术相比，采用亚肺叶切除术治疗的患者生存期更长，消融术和亚肺叶切除术两组之间存在显著性差异，因此，对于Ia期NSCLC患者，若能够耐受手术，更推荐采用亚肺叶切除术治疗。
